# The Association between Peri-Transplant RBC Transfusion and Graft Failure after Kidney Transplantation: A Nationwide Cohort Study

**DOI:** 10.3390/jcm10163750

**Published:** 2021-08-23

**Authors:** Kyungho Lee, Seohee Lee, Eun Jin Jang, Ga Hee Kim, Seokha Yoo, Minkyoo Lee, Hye Ryoun Jang, Ho Geol Ryu

**Affiliations:** 1 Division of Nephrology, Department of Medicine, Samsung Medical Center, Sungkyunkwan University School of Medicine, Seoul 06351, Korea; kidney.kh@gmail.com; 2 Department of Anesthesiology and Pain Medicine, Seoul National University Hospital, Seoul National University College of Medicine, Seoul 03080, Korea; leesen34@gmail.com (S.L.); muroki22@gmail.com (S.Y.); 2016.real@gmail.com (M.L.); 3 Department of Information Statistics, Andong National University, Andong 36729, Korea; jejstat@gmail.com; 4 Department of Statistics, Kyungpook National University, Daegu 41566, Korea; genius9105@naver.com

**Keywords:** kidney transplantation, red blood cells, transfusion, graft failure, survival

## Abstract

Background: Patients undergoing kidney transplantation (KT) often receive red blood cell (RBC) transfusion during admission for KT which may increase the risk of allosensitization. The association between peri-transplant RBC transfusion and graft survival was evaluated using a nationwide cohort. Methods: This retrospective study analyzed 13,871 patients who underwent KT in Korea between 2007 and 2015. The outcomes were graft failure rate and overall patient survival depending on the amount of RBC transfusion. Results: The overall graft failure rate was 15.5%. Compared to the graft failure rate of 13.5% in the no transfusion group, the graft failure rate was 15.4% in the 1–2 units group (sHR 1.06 (95% CI 0.97–1.17), *p* = 0.216), 21.4% in the 3–5 units group (sHR 1.39 (1.21–1.61), *p* < 0.001), and 35.3% in the 6 or more units group (sHR 2.20 (1.70–2.85), *p* < 0.001). The overall survival rate was 97.5% in the no transfusion group, compared to 95.9% in the 1–2 units group (HR 1.50 (1.22–1.83), *p* < 0.001), 92.0% in the 3–5 units group (HR 2.43 (1.87–3.15), *p* < 0.001), and 67.5% in the 6 or more units group (HR 6.81 (5.03–9.22), *p* < 0.001). Conclusions: Peri-transplant RBC transfusion was independently associated with the increased risk of renal allograft failure and death in KT patients.

## 1. Introductions

Kidney transplantation (KT) is the treatment of choice for end-stage renal disease (ESRD) with clear advantages over renal replacement therapy in survival and quality of life [1]. Graft survival after KT has significantly improved with recent 5-year graft survival rates ranging between 75% and 90% [1,2,3]. However, there seems to be room for improvement in long-term graft survival rates [4].

Blood transfusion prior to KT was common practice [5] until the early 1980s with intentions to improve graft survival rates through immunosuppression by transfused white blood cells [6]. Following increased graft survival due to improved human leukocyte antigen (HLA) matching and more effective immunosuppressive agents, the benefit of peri-transplant transfusion in KT became insignificant [7]. In the 1990s, most KT centers moved away from routine pre-transplant blood transfusion, especially for immunosuppression purposes [8]. In addition, anemia in patients undergoing KT became less frequent with the introduction of erythropoiesis-stimulating agents (ESAs), which are now universally used [9]. Furthermore, blood transfusion in KT recipients can lead to sensitization, which may increase the possibility of antibody formation that may attack the allograft [10].

Recent data show that 52–64% of patients undergoing KT still receive blood transfusion [10,11]. However, the impact of peri-transplant transfusion on the renal allograft outcome remains unclear. Studies evaluating the relationship between peri-transplant transfusion and the renal allograft outcome have shown conflicting results [10,11]. To evaluate the association between peri-transplant red blood cell (RBC) transfusion and graft survival, a nationwide cohort study using the National Healthcare Insurance Service (NHIS) database was conducted.

## 2. Materials and Methods

This study was a retrospective cohort study and the study protocol conformed to the ethical guidelines of the 1975 Declaration of Helsinki as reflected in a priori approval by the Institutional Review Board of Seoul National University Hospital (1708-061-877). The clinical and research activities reported are consistent with the Principles of the Declaration of Istanbul as outlined in the ‘Declaration of Istanbul on Organ Trafficking and Transplant Tourism’.

### 2.1. Data Source and Study Population

The NHIS database contains all claims data for more than 97% of the population who are covered under the National Healthcare Insurance (NHI) program and the Medical Aid program in Korea. The NHIS database is provided to researchers after de-identification for research purposes and generation of real-world evidence.

Adult patients (age ≥ 19) who received KT between 2007 and 2015 were identified from the NHIS database by searching the NHI procedure code for KT during hospitalization. Patients with simultaneous NHI procedure codes for other solid organ transplantations such as pancreas, liver, or heart transplantation during the same admission for KT were excluded. After identification of adult KT recipients, underlying comorbidities including hypertension, diabetes mellitus, coronary artery disease, and cardiovascular disease were extracted from the database using ICD-10 (International Classification of Diseases, 10th revision) codes. To adjust for the severity of illness, the Elixhauser Comorbidity Index, derived from 30 disease entities using ICD-10 codes and shown to correlate with hospital mortality, was used as a covariate.

The two outcomes of interests were allograft failure (not including death with function) and all-cause mortality. Allograft failure was detected by NHI procedure codes for dialysis or retransplantation. Long-term mortality was detected when healthcare coverage by the NHI was terminated based on automatically reported death certificates to the NHI. In-hospital mortality, intensive care unit (ICU) length of stay, and hospital length of stay were also extracted. To analyze immunologic risk factors, the following data were extracted from the database: presence of donor specific antibody (DSA; defined by NHI procedure code for desensitization therapy), regimens of induction treatment, delayed graft function (DGF; defined as the requirement for dialysis within the first week after transplantation), and acute allograft rejection (defined by NHI procedure code for anti-rejection therapy). The data on perioperative bleeding complications were obtained from the database using the NHI procedure codes for angiographic embolization and bleeding control operation.

### 2.2. Statistical Analysis

Patient characteristics were compared according to the number of transfused packed RBC units using the chi-square test or Fisher’s exact test.

A competing-risk analysis model (Fine and Gray model) was used considering death with functioning graft as a competing event to investigate the association between transfused RBC units and graft failure with adjustment of multiple covariates. The competing-risk analysis results were presented as a subdistribution hazard ratio (sHR) with a 95% confidence interval (CI). To analyze the association between transfused RBC units and mortality, a Cox proportional hazards model was used and the results were presented as a hazard ratio (HR) with a 95% CI.

The Kaplan–Meier survival curve after KT depends on the number of transfused RBC units and performed the log-rank test to compare the survival curve. All analyses were performed using SAS 9.4 (SAS Institute, Cary, NC, USA). Results were considered statistically significant when two-sided *p*-values were less than 0.05.

## 3. Results

A total of 13,781 KTs were performed from 2007 to 2016 in Korea. Patient and center characteristics are presented in Table 1. All patients were divided into four groups depending on the number of transfused packed RBC units during the hospitalization period for KT. A total of 6594 patients did not receive RBC transfusion during the perioperative period. Of the patients who received transfusion, 5687 patients received 1–2 units, 1212 patients received 3–5 units, and 378 patients received 6 units or more. The proportions of positive-DSA were significantly higher in patients who received larger amounts of RBC transfusion (Table 1).

The overall graft failure rates at 1, 3, 5, and 7 years after KT were 7.0% (811/11,665), 14.1% (1150/8163), 18.3% (904/4942), and 21.3% (507/2381), respectively. There was a positive correlation between the number of RBC transfusions and graft failure rates at various time points after KT (Table 2). 

**Figure 1:**
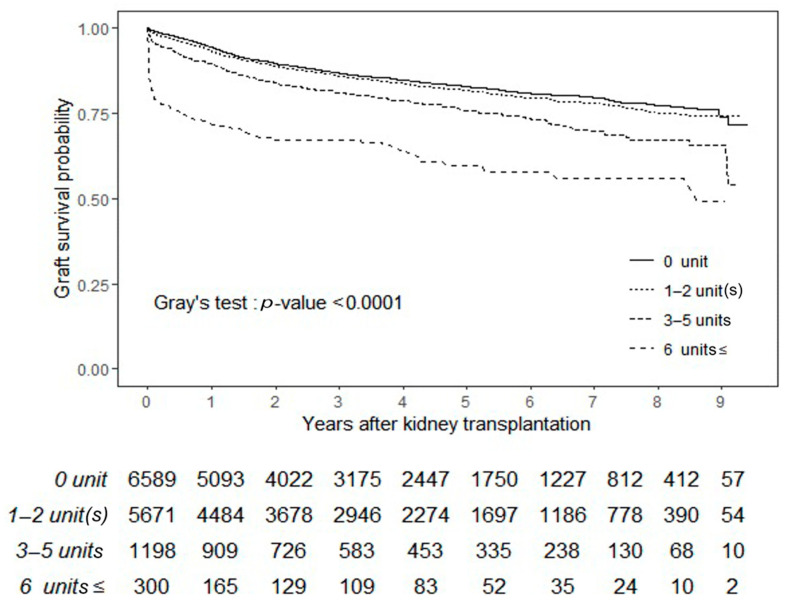
Death censored graft survival rate according to the amount of perioperative RBC transfusion. Graft survival rates were higher in patients who received less RBC transfusion (Fine and Gray model, *p* < 0.001). Abbreviations: KT, kidney transplantation and RBC, red blood cell.

The competing-risk analysis for graft failure treating death with functioning graft as a competing event showed that the risk of graft failure was significantly higher in patients who received larger amounts RBC transfusion. Patients who received 6 or more units (sHR 2.20 (95% CI: 1.70, 2.85)) and patients who received 3–5 units (sHR 1.39 (95% CI: 1.21, 1.61)) showed higher risk of graft failure compared to the patients who did not receive transfusion. The risk of graft failure after KT in patients who received 1–2 units was comparable to patients who did not receive transfusion (sHR 1.06 (95% CI: 0.97, 1.17)) (Table 3). Deceased donor KT, DSA positivity, acute rejection, and delayed graft function were also independently associated with a higher risk of graft failure. Patients who received induction therapy with anti-thymocyte globulin or basiliximab showed a higher risk of graft failure compared to patients who only received methylprednisolone. Death censored graft survival rates according to the amount of RBC transfusion are presented in Figure 1 (*p* < 0.001).

**Figure 2:**
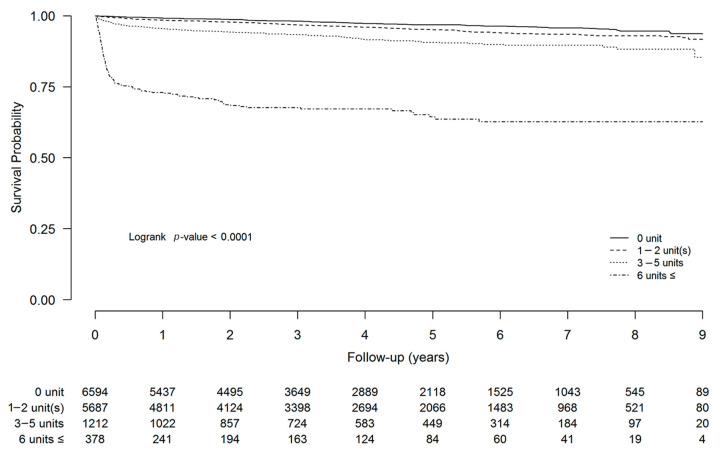
Patient survival according to the amount of perioperative RBC transfusion. Long-term survival analysis of up to 10 years after KT showed higher survival rates in patients who received less RBC transfusion (log-rank test *p* < 0.001). Abbreviations: KT, kidney transplantation and RBC, red blood cell.

In the Cox proportional hazard model for all-cause mortality, adjusted HRs were higher in patients who received 6 or more units of RBC compared to patients who received 5 units or less (Table 4). Deceased donor KT, acute rejection, and delayed graft function were also associated with higher mortality. The long-term survival analysis of up to 10 years showed a higher probability of survival in patients who received less RBC transfusion (log-rank test *p* < 0.001, Figure 2).

## 4. Discussion

Our results suggest that 3 or more units of RBC transfusion during the perioperative period for KT may be associated with an increased risk of graft failure after KT. The association between perioperative RBC transfusion and renal allograft survival in a nationwide large cohort may suggest the importance of adequate management for anemia in ESRD patients waiting for KT. 

As the incidence and prevalence of chronic kidney disease (CKD) grows, the number of patients on the waiting list for KT is also increasing. Death-censored kidney allograft survival has increased steadily over the past decade in both adult and pediatric patients [12]. The Scientific Registry of Transplant Recipients reported a 10-year overall graft survival in both living and deceased donors of approximately 70 to 80%, which indicated significant progress from the previous 35 to 40% [1,2]. Our data showed a similar 5-year renal allograft survival of 82%.

Many factors affect the long-term outcome of KT, which is often defined as patient death or renal dysfunction leading to graft loss requiring dialysis [13]. Donor age and HLA matching were well-known prognostic factors of the renal allograft outcome [14,15]. Key recipient factors include age, disease recurrence, HLA matching, HLA immunization, ethnic background, time on dialysis, and cardiovascular comorbidities [13]. To analyze the association between the RBC transfusion, renal outcome, and patient survival after KT, multivariable analyses were performed by adjusting for well-known prognostic factors such as age, donor type, and major comorbidities.

The NHI program is the universal healthcare coverage system in Korea. The NHIS provides healthcare insurance to more than 97% of the population in Korea through the NHI program and is the single payer of the NHI program. The remaining 3% of the population with the lowest income are supported by the Medical Aid program [16]. The broad inclusiveness and completeness of the database is one of the main strengths of our study. In addition, concrete outcomes such as graft failure and mortality were used as end points.

Studies prior to the 1980s suggested that pre-transplant RBC transfusion may improve allograft survival [17,18]. Based on these findings, at the time many transplant centers routinely transfused patients prior to KT [5]. Subsequently, the practice of pre-transplant RBC transfusion decreased as the risk of immune sensitization and potential infection outweighed the benefits of transfusion, especially with advances in immunosuppressant drugs, leading to improved outcomes without pretransplant transfusion [19]. The benefit of the restrictive transfusion strategy has been shown in various conditions due to the association between liberal transfusion strategies and adverse outcomes including infection, acute respiratory distress syndrome, multi-organ dysfunction, and mortality [6].

The impact of transfusion in KT has not been studied extensively and the results of the few studies that evaluated the impact of transfusion on renal allograft outcomes were inconsistent. Transfusion prior to KT was recently reported to be associated with the development of anti-HLA antibodies in patients with no previous organ transplantation or pregnancy [20]. In contrast, although analyzed using a less sensitive assay for antibody detection, transfusion in patients with a functioning graft under maintenance immunosuppression was not associated with the de novo formation of HLA antibodies [11]. However, one recent study using modern sensitive immunological tests for antibody detection demonstrated that overall incidences of DSAs and antibody-mediated rejection were significantly higher in KT patients who received transfusion after transplantation [10].

Although, our study focused on the effect of perioperative RBC transfusion on the renal outcome, transfusion after discharge from the admission for KT may have affected allograft outcomes. However, previous studies have shown that most blood transfusion (70 to 90%) occurred during the perioperative hospital admission period [10,11]. In both previous studies and our study, most patients received less than 3 units of RBCs [10,11,21]. In addition, considering that it takes approximately 28 days for the renal allograft to produce erythropoietin after KT [22] and that DGF or chronic allograft dysfunction may cause the suppression of erythropoietin production, transfusion long after KT is likely to be a consequence of graft dysfunction rather than a cause. 

Our data suggests that perioperative transfusion may have negative effects on long-term graft survival after kidney transplantation. The Renal Association Clinical Practice Guidelines suggest that hemoglobin levels should be maintained in the range of 10–12 g/dL in CKD patients with ESA therapy [23]. Maintaining the hemoglobin level preoperatively with optimal ESA therapy could be the first step to minimize perioperative blood transfusion. Novel drugs such as hypoxia-inducible factor stabilizers, maintaining normothermia for optimal hemostasis, and the application of modern cell salvage technologies for rapid autologous transfusion may be additional strategies to avoid allogenic blood transfusion. Avoiding unnecessary blood tests during the perioperative period may also contribute to minimizing transfusion [24].

Patients undergoing deceased donor KT recipients showed a higher transfusion rate compared to living donor KT recipients (63% vs. 50%), which is consistent with the findings of previous studies [11]. Given the unpredictable timing of deceased donor organs, achieving and maintaining optimal hemoglobin levels before KT may have been difficult in deceased donor KT. An additional explanation may be that the discontinuation of antiplatelet therapy prior to KT may not be feasible in the deceased donor KT setting [25]. Considering the higher risk of DGF in deceased donor KT [26], especially with extended criteria donors [27], prolonged insufficient erythropoietin production of DGF may have increased the need for transfusion after transplantation.

This study has several limitations that requires consideration. First, due to the nature of administrative data, the NHIS database lacks several clinically relevant variables including laboratory data and detailed clinical information. Although data regarding perioperative bleeding complications were added to compensate for the lack of hemoglobin levels, comparing patients with similar hemoglobin levels may have provided a clearer picture. However, we believe that the clinical implications of our study are still considerable as specific clinically relevant outcomes were analyzed, adjusting for major comorbidities and immunologic factors. Second, data regarding the types of transfused RBCs such as leukocyte depleted or radiated RBCs were not available in our analyses. Although leukocyte depleted RBCs are commonly used to prevent alloimmune sensitization, HLA class I molecules are expressed constitutively on erythrocytes even though their expression levels are low. The content of HLA molecules within leukocyte depleted RBCs is sufficient to induce HLA class I sensitization. Leukodepletion of blood products is not sufficient to prevent the risk of allosensitization [28,29]. Third, institutional differences in surgical techniques and RBC transfusion practices might also have affected the results as confounders. Recognizing that protocols for KT and transfusion have been established and standardized by several leading transplantation centers, the impact of the variation may be considered as minimal. Fourth, the underlying diseases causing allograft failure could not be identified using the NHIS database. Both alloimmune mechanisms and non-alloimmune injuries such as recurrent primary diseases contribute to allograft failure [30]. Alloimmune responses are known to play substantial roles in the progression of allograft failure even in patients with recurrent primary disease [31]. Although the main cause of graft failure was not available in our study, alloimmune processes provoked by RBC transfusion may have contributed to graft failure. However, future studies using histologic data and serial serum DSA levels are required to confirm the long-term immunological effects of RBC transfusion.

Despite these limitations, our study provides some important clinical implications. Our study is the first large scale study to investigate the relationship between perioperative RBC transfusion and graft survival after KT. In addition, selection bias was minimized by using the NHIS database, which includes nearly all KT operations performed in the recent 10 years in Korea. In conclusion, KT patients who received more RBC transfusion over 2 units during the perioperative period showed lower graft survival compared to those who received no transfusion or less than 3 units.

## Figures and Tables

**Table 1:** Patient characteristics according to the amount of RBC transfusion.

		Units of Transfused Red Blood Cells				
	Total (*n* = 13,871)	0 Units (*n* = 6594)	1–2 Units (*n* = 5687)	3–5 Units (*n* = 1212)	6 or More Units (*n* = 378)	*p-*Value
Age						
19–49	7524 (54.2)	3693 (56.0)	3064(53.9)	614 (50.7)	152 (40.2)	<0.001
50–59	4502 (32.5)	2102 (31.9)	1845 (32.4)	407 (33.6)	148 (39.2)	
≥ 60	1846 (13.3)	799 (12.1)	778 (13.7)	191 (15.8)	78 (20.6)	
Sex						
Female	5642 (40.7)	2286 (34.7)	2600 (45.7)	571 (47.1)	185 (48.9)	
Male	8229 (59.3)	4308 (65.3)	3087 (54.3)	641 (52.9)	193 (51.1)	<0.001
Comorbidities						
Hypertension	6765 (48.8)	3281 (49.8)	2709 (47.6)	581 (47.9)	194 (51.3)	0.067
Diabetes mellitus	3879 (28.0)	1798 (27.3)	1554 (27.3)	393 (32.4)	134 (35.4)	<0.001
Coronary artery disease	2848 (20.5)	1395 (21.2)	1076 (18.9)	269 (22.2)	108 (28.6)	<0.001
Cerebrovascular disease	800 (5.8)	339 (5.1)	342 (6.0)	81 (6.7)	38 (10.1)	0.002
Deceased donor transplantation	3077 (22.2)	1153 (17.5)	1363 (24.0)	379 (31.3)	182 (48.1)	<0.001
Positive donor specific antibody	2084 (15)	624 (9.5)	959 (16.9)	336 (27.7)	165 (43.7)	<0.001
Induction treatment						
Methylprednisolone	481 (3.5)	250 (3.8)	187 (3.3)	36 (3.0)	8 (2.1)	<0.001
Anti-thymocyte globulin	1897 (13.7)	829 (12.6)	745 (13.1)	226 (18.6)	97 (25.7)	
Basiliximab	11,405 (82.2)	5479 (83.1)	4711 (82.8)	945 (78.0)	270 (71.4)	
Rituximab	88 (0.6)	36 (0.5)	44 (0.8)	5 (0.4)	3 (0.8)	
Acute rejection	1096 (7.9)	571 (8.7)	412 (7.2)	94 (7.8)	19 (5.0)	0.005
Delayed graft function	253 (1.8)	18 (0.3)	60 (1.1)	48 (4.0)	127 (33.6)	<0.001
Elixhauser Comorbidity Index	14.1 (8.6)	14.1 (8.4)	13.7 (8.6)	15.0 (9.0)	17.5 (9.8)	<0.001

Data are presented as a number (percentage) or mean (standard deviation).

**Table 2:** Graft failure rate, in-hospital mortality, and perioperative bleeding events according to the amount of transfusion.

	Total (*n* = 13,871)	Units of Transfused Red Blood Cells				
		0 Units (*n* = 6594)	1–2 Units (*n* = 5687)	3–5 Units (*n* = 1212)	6 or More Units (*n* = 378)	*p*-Value
1-year graft failure rate	811/11,66 (7.0)	307/5477 (5.6)	313/4870 (6.4)	115/1057 (10.9)	76/261 (29.1)	<0.001
3-year graft failure rate	1150/8163 (14.1)	476/3708 (12.8)	461/3498 (13.2)	149/768 (19.4)	64/189 (33.9)	<0.001
5-year graft failure rate	904/4942 (18.3)	362/2176 (16.6)	376/2168 (17.3)	127/494 (25.7)	39/104 (37.5)	<0.001
7-year graft failure rate	507/2381 (21.3)	221/1081 (20.4)	205/1040 (19.7)	61/207 (29.5)	20/53 (37.7)	<0.001
In-hospital mortality	113/13,871 (0.8)	5/6594 (0.1)	16/5689 (0.3)	14/1212 (1.2)	78/376 (20.6)	<0.001
Bleeding control operation	331/13,871 (2.4)	26/6594 (0.4)	94/5687 (1.7)	95/1212 (7.8)	116/378 (30.7)	<0.001
Angiographic embolization	81/13,871 (0.6)	5/6594 (0.1)	21/5687 (0.4)	16/1212 (1.3)	39/378 (10.3)	<0.001

Data are presented as a number (percentage).

**Table 3:** Competing-risk analysis for graft failure (competing event of death with functioning graft).

	Univariate Analyses			Multivariable Analyses		
	Hazard Ratio	95% CI	*p-*Value	Hazard Ratio	95% CI	*p-*Value
Age						
19–49						
50–59	1.09	0.99, 1.20.	0.074	1.08	0.98, 1.18.	0.132
≥60	1.09	0.96, 1.25.	0.197	1.04	0.90, 1.19.	0.620
Sex						
Female						
Male	0.96	0.88, 1.04.	0.303	0.97	0.89, 1.06.	0.549
Living donor kidney transplantation						
Deceased donor kidney transplantation	1.81	1.66, 1.98.	<0.001	1.68	1.53, 1.85.	<0.001
Donor specific antibody						
Absent						
Present	1.28	1.13, 1.44.	<0.001	1.17	1.03, 1.34.	0.017
Induction treatment						
Methylprednisolone (reference)						
Anti-thymocyte globulin	2.67	2.01, 3.55.	<0.001	2.02	1.52, 2.68.	<0.001
Basiliximab	1.74	1.33, 2.27.	<0.001	1.42	1.09, 1.84.	0.009
Rituximab	3.17	1.27, 7.93.	0.014	2.74	1.12, 6.75.	0.028
Acute rejection						
Absent						
Present	2.93	2.65, 3.25.	<0.001	2.97	2.67, 3.31.	<0.001
Delayed graft function						
Absent						
Present	3.24	2.49, 4.20.	<0.001	1.94	1.44, 2.62.	<0.001
Transfused red blood cell units						
None						
1–2 units	1.09	1.00, 1.20.	0.061	1.06	0.97, 1.17.	0.216
3–5 units	1.57	1.37, 1.81.	<0.001	1.39	1.21, 1.61.	<0.001
6 units or more	3.36	2.69, 4.20.	<0.001	2.20	1.70, 2.85.	<0.001
Elixhauser Comorbidity Index	1.00	1.00, 1.01.	0.151	1.00	1.00, 1.01.	0.511

Abbreviation: CI, confidence interval. The subdistribution hazard of ESRD (end-stage renal disease) was used for the hazard ratio (competing event for death with functioning graft).

**Table 4:** Cox regression analysis for death after kidney transplantation.

	Univariate Analyses			Multivariable Analyses		
	Hazard Ratio	95% CI	*p-*Value	Hazard Ratio	95% CI	*p-*Value
Age						
19–49						
50–59	2.59	2.14, 3.14.	<0.001	2.31	1.91, 2.80.	<0.001
≥ 60	5.27	4.29, 6.47.	<0.001	4.33	3.51, 5.34.	<0.001
Sex						
Female						
Male	1.24	1.05, 1.46.	0.010	1.25	1.06, 1.48.	0.009
Living donor kidney transplantation						
Deceased donor kidney transplantation	2.23	1.90, 2.62.	<0.001	1.55	1.31, 1.84.	<0.001
Donor specific antibody						
Absent						
Present	1.43	1.15, 1.76.	0.001	0.93	0.74, 1.16.	0.504
Induction treatment						
Methylprednisolone (reference)						
Anti-thymocyte globulin	2.68	1.57, 4.54.	<0.001	1.28	0.75, 2.19.	0.367
Basiliximab	1.70	1.03, 2.79.	0.038	1.23	0.74, 2.03.	0.419
Rituximab						
Acute rejection						
Absent						
Present	1.53	1.21, 1.94.	<0.001	1.74	1.37, 2.21.	<0.001
Delayed graft function						
Absent						
Present	15.05	12.17, 18.62.	<0.001	4.15	3.14, 5.48.	<0.001
Transfused red blood cell units						
None						
1–2 units	1.55	1.27, 1.89.	<0.001	1.50	1.22, 1.83.	<0.001
3–5 units	3.07	2.39, 3.94.	<0.001	2.43	1.87, 3.15.	<0.001
6 units or more	16.49	13.06, 20.83.	<0.001	6.81	5.03, 9.22.	<0.001
Elixhauser Comorbidity Index	1.04	1.03, 1.05.	<0.001	1.02	1.01, 1.03.	<0.001

Abbreviation: CI, confidence interval.

## Data Availability

Restrictions apply to the availability of these data. Data were obtained from National Health Insurance Service (NHIS) and are available from the authors with the permission of NHIS.

## Abbreviations

CI	confidence interval
CKD	chronic kidney disease
DDKT	deceased donor kidney transplantation
DGF	delayed graft function
DSA	donor specific antibody
ESA	erythropoiesis-stimulating agent
ESRD	end-stage renal disease
HLA	human leukocyte antigen
HR	hazard ratio
ICU	intensive care unit
KT	kidney transplantation
NHI	National Healthcare Insurance
NHIS	National Healthcare Insurance Service
OR	odds ratio
RBC	red blood cell
sHR	subdistribution hazard ratio
